# Contribution of Single-Fiber Evaluation on Monitoring Outcomes Following Injection of Botulinum Toxin-A: A Narrative Review of the Literature

**DOI:** 10.3390/toxins13050356

**Published:** 2021-05-17

**Authors:** Hélène Moron, Corine Gagnard-Landra, David Guiraud, Arnaud Dupeyron

**Affiliations:** 1 Department of Functional Exploration of the Nervous System and Acupuncture, CHU Nîmes, Univ Montpellier, 30029 Nîmes, France; corinne.gagnard.landra@chu-nimes.fr; 2 EuroMov DHM, IMT Ales, Univ Montpellier, 34090 Montpellier, France; arnaud.dupeyron@umontpellier.fr; 3 CAMIN, INRIA, Univ Montpellier, 34090 Montpellier, France; david.guiraud@inria.fr; 4 Department of Physical and Rehabilitation Medicine, CHU Nîmes, Univ Montpellier, 30029 Nîmes, France

**Keywords:** botulinum toxin, single-fiber, jitter, neuromuscular junction, electromyography

## Abstract

Botulinum toxin-A (BoNT-A) blocks acetylcholine release at the neuromuscular junction (NMJ) and is widely used for neuromuscular disorders (involuntary spasms, dystonic disorders and spasticity). However, its therapeutic effects are usually measured by clinical scales of questionable validity. Single-fiber electromyography (SFEMG) is a sensitive, validated diagnostic technique for NMJ impairment such as myasthenia. The jitter parameter (µs) represents the variability of interpotential intervals of two muscle fibers from the same motor unit. This narrative review reports SFEMG use in BoNT-A treatment. Twenty-four articles were selected from 175 eligible articles searched in Medline/Pubmed and Cochrane Library from their creation until May 2020. The results showed that jitter is sensitive to early NMJ modifications following BoNT-A injection, with an increase in the early days’ post-injection and a peak between Day 15 and 30, when symptoms diminish or disappear. The reappearance of symptoms accompanies a tendency for a decrease in jitter, but always precedes its normalization, either delayed or nonexistent. Increased jitter is observed in distant muscles from the injection site. No dose effect relationship was demonstrated. SFEMG could help physicians in their therapeutic evaluation according to the pathology considered. More data are needed to consider jitter as a predictor of BoNT-A clinical efficacy.

## 1. Introduction

Botulinum toxin type A (BoNT-A) has been approved by the U.S. Food and Drug Administration and the European regulatory agencies as an effective, validated and safe treatment for neuromuscular disorders such as involuntary spasms including blepharospasm (BSP) [1], hemifacial spasm (HSF) [2], dystonic like cervical dystonia [3] and focal spasticity, especially in stroke survivors [4,5,6] and for non-therapeutic cosmetic purposes [7]. However, there is not yet a general consensus on the best way to evaluate its efficacy. For involuntary spasm pathologies, clinical scales are used, for example the Frequency of Involuntary Movements score and the Severity Rating Scale. For dystonia, the Toronto Western Spasmodic Torticollis Rating Scale is the most widely used and disseminated clinical scale according to a Cochrane review [3]. The Facial Grading System is employed in HSF assessment and is based on the evaluation of resting symmetry, degree of voluntary excursion of facial muscles, and degree of synkinesis associated with specified voluntary movement [8,9]. Instrumental scales have been proposed for the evaluation of severity and synkinesis improvement, such as a machine learning approach for automated facial measurements [10,11,12].

For spasticity, clinical scales such as the Ashworth scale [13,14], the modified Ashworth scale (MAS) [15], the Tardieu scale [16], and the modified Tardieu scale [16,17,18,19,20] are used to measure the response to a passive movement [13,21,22] induced by BoNT-A. All these scales are based on ordinal, predetermined values, which are subject to the evaluator’s interpretation. Several studies have queried their pertinence [15,23,24], validity and objectivity [15,20,25,26,27]. For example, the MAS is fast, easy-to-use, with satisfactory intra-evaluator reliability, especially in the upper limbs, but its inter-rater reliability remains questionable [23,28]. Therefore, it seems necessary to develop objective scales [29].

Several instrumental [20,30,31,32] and non-instrumental [33,34,35] scales have also been proposed, including some designed to objectively quantify muscle tone alterations in spasticity and their modifications induced by BoNT-A injection. Indeed, some describe mechanical and structural properties via ultrasound imaging [36,37] and MRI [38], viscoelastic properties via elastography [39,40,41,42], and Myoton^®^ technology. Others report mechanical properties via mechanography and electrophysiological properties via electromyography (EMG) such as the M response and the H and F waves [43,44,45,46,47,48,49,50] induced by a peripheral nerve electrical stimulus. However, the reproducibility of these measurements depends on recording conditions (electrode site, position of the joint) and thus requires strict standardization. Therefore, to date, there is no recommendation for any objective measure in practice. 

One solution could be the single-fiber EMG (SFEMG) which was first described in 1963 by Stålberg and Ekstedt, and is considered to be the most sensitive test for diagnosing NMJ disorders [51,52,53] such as myasthenia, especially ocular myasthenia [54]. Moreover, SFEMG may help to identify conduction impairment in atypical forms of NMJ disorders before a complete conduction block has occurred, for which conventional repetitive stimulation tests are imperfect [55,56]. SFEMG allows selection of two muscle fibers from the same motor unit and records their electrophysiological activity, measuring the interpotential intervals, e.g., the time between two activated single muscle fibers’ potential and their modification (jitter measuring, Supplementary File). This interval, the jitter, is automatically estimated by an electromyograph and results mostly from neuro-muscular transmission. Jitter is measured from 100 consecutive discharges of approximately 20 pairs of activated single muscle fibers. Standard criteria usually used are the peak-to-peak amplitude > 200 µV, rise time < 300 µs and constant shape over time. Jitter is calculated as the mean consecutive difference (MCD). The time between two activated single muscle fibers’ potential, the IPI (interpotential interval), is measured by subtracting the IPI from each discharge from the IPI of the next discharge and summing the differences; the sum is then divided by the number of total discharges (n) minus one, according to the following formula:

MCD= [|IPI1 − IPI2| + |IPI2 − IPI3| + |…| + |IPIn-1 − IPIn|]/n − 1(1)

Jitter variations are largely influenced by the release of acetylcholine (ACh) within the motor end plate [57]. Thus, any NMJ dysfunction will lengthen the jitter. Indeed, the norms for jitter vary according to several parameters, e.g., methodology used [58] (stimulated or voluntary SFEMG) and patient characteristics (age, muscles considered), resulting in various suggested stratified reference values [59,60,61,62,63]. Currently, a wider and cheaper application of SFEMG is the standardization of this technique with concentric needle electrode (CNE) [64,65]. International experts have established guidelines for SFEMG for both standard electrode and CNE, including optimal condition of measurement and analysis of the signal [66]. However, protocols differ in activation or voluntary mode which questions the pertinence of these reference values.

The purpose of this work was to present a review of the literature of the value of jitter to characterize electrophysiological changes induced by BoNT-A, related to local and remote effects. We also aimed to confirm whether there was any association between jitter and clinical signs, and the treatment procedure (dose and timing). Finally, we wished to make suggestions for future research in this area.

## 2. Results

### 2.1. Selection of the Studies

**Figure 1:**
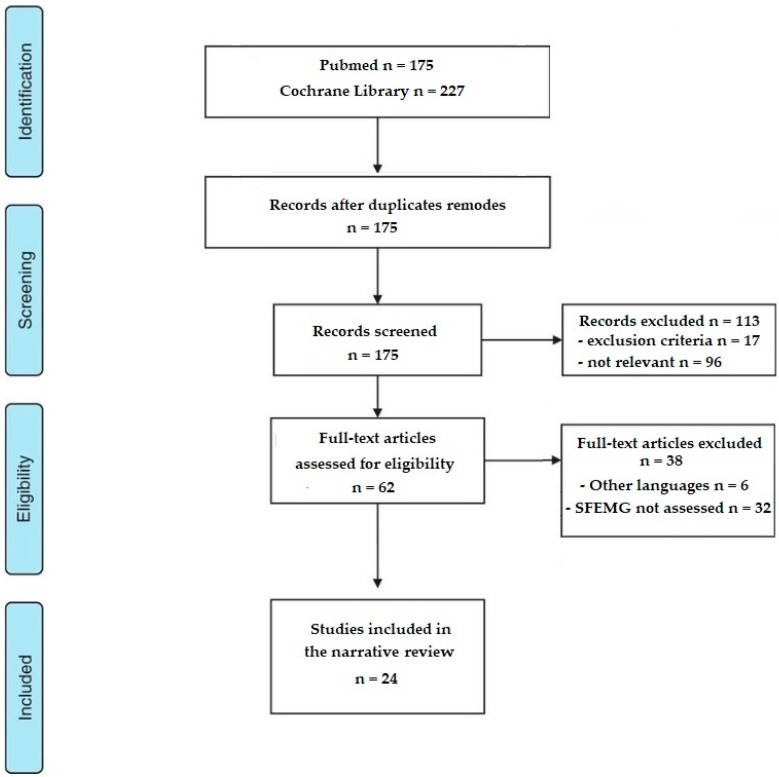
Study flow chart.

Figure 1 shows the flowchart of study retrieval, screening and eligibility assessment. The literature search yielded 175 potentially eligible articles from the initial search in Pubmed and 227 in Cochrane Library. When duplicates were eliminated, 175 records remained. After screening abstracts, 113 articles were removed for lack of relevance (n = 96) or for not meeting inclusion criteria (n = 17). These included six case reports about foodborne botulism [67,68,69,70,71,72], one on infant botulism [73] and one on wound botulism [74]. Only cases of iatrogenic botulism secondary to the use of BoNT-A treatment were analyzed. Five systematic or narrative reviews on the clinical spectrum of botulism [75], clinical impact of SFEMG [76,77] and botulinum toxin in neurological disease [78,79] were excluded after reading and selecting references of interest (20 references). Finally, four animal studies were excluded [80,81,82,83].

Among the 62 articles selected as being potentially eligible, 38 were excluded after analysis of the full text: six articles were withdrawn because the article was written in a language other than French or English: Japanese [84,85], Chinese [86] and Russian [87], Italian [88] and German [89]; and 32 dealing with other electrophysiological techniques without the use of SFEMG. 

Originally, only studies with high quality data (i.e., randomized control trials (RCT) and prospective studies with robust methodology) were planned to be retained, but due to the sparsity of relevant studies, the filter was voluntarily broadened to include all clinical trials of inferior methodological quality, as well as those concerning fields of application for BoNT-A other than spasticity (dystonia, blepharon and facial spasms, dysphonia, esthetic and surgical applications). From the review of Leonardi et al. [90], we identified correspondence, communications and letters to the editor which were not found in the initial search because of SFEMG missing key words (Tugnoli [91], Emmerson [92], Coban [93]). These articles were not included in this narrative review due to the lack of data on jitter value (only judged “abnormal” or “normal”). Finally, 24 articles (either with standard electrode or CNE) from 1986 to 2020 were retained: 12 case reports, six RCT, six observational studies (three retrospective and three prospective). Table 1 lists the main results and Table S2 in Supplementary Materials the detailed ones.

### 2.2. Literature Analysis

#### 2.2.1. Local Effects of BoNT-A

Only six studies explored the results of SFEMG in the injected muscle with clinical evolution; in orbicularis oculi (OO) [94,100,101,115], extensor digitorum brevis (EDB) [113] and abductor digiti minimi (ADM) [116] muscles. Doses, timing of recordings and jitter results are detailed in Table 1 and Table S2 in the Supplementary Materials. Initially normal [97,100,101,109,113,116], the jitter was shortened following injection, to the shortest within 24 h, with a maximum deviation of three times the pre-injection level [94]. Most commonly, the jitter lengthened before Day 7 [94,100] until Day 15 [100,101,116], with peak values ranging between 3 [94,100,101,116] and 10 times the pre-injection level [94]. However, some patients showed peaks at 2 months at twice the pre-injection level [113]. The decrease was generally measured over 2 months [104], but did not seem to fully recover within 3 months [94,100,101,104,113,115]. Sometimes jitter remained over twice the pre-injection level [113], even at 6 months [101], without any explanation or theories provided by the authors [94]. Notably, the definition of normal values before injection was not always clear (Table S1 in Supplementary Materials). 

#### 2.2.2. Distant Effects of BoNT-A

Increased jitter is found both in the muscles in the immediate vicinity and more remote from the point of injection, raising the possibility of propagation, diffusion and migration of BoNT-A. When cervical muscles [95,96,97,98,99] or OO [94,98] were injected, the extensor digitorum communis (EDC), biceps brachial (BB) and tibialis anterior (TA) muscles showed longer pathologic jitter. 

In the case of dystonia with BoNT-A injection in different cervical muscles, from baseline normal value for some patients (Table 1, Tables S1 and S2 in Supplementary Materials), the BB jitter [96] increased by 31% from a mean value of 28.9 µs +/− 10.9 µs to 88 µs (standard deviation unspecified) by the last assessment performed at 4 to 12 weeks (Table 1 and Table S2 in Supplementary Materials); the EDC jitter [95,99] lengthened with a median onset of changes at 7 Day [99], or later at around 2 weeks [95] for a first SFEMG, with maximum pathologic results observed a median of 37 days after injection [99] (Table 1 and Table S2 in Supplementary Materials) and a decrease without normalization for the only patient analyzed at 4 months [95]. Jitter stayed normal in EDC muscle throughout the study in the placebo group (*p* < 0.05), contrary to the BoNT-A group whose pre-treatment normal jitter lengthened and decreased respectively at 2 and 12 weeks post-injection with full recovery (Table S2 in Supplementary Materials) for half the patients [97].

For patients with BSP treated with injection of the OO muscle, jitter increased both in the neighboring muscles (frontalis muscle) and at distance (EDC and BB muscles) [94]. In addition, jitter also increased on the contro-lateral placebo-side OO [100], although to a lesser extent (maximum value multiplied by 1.6 baseline value at 2 weeks) and with an earlier decrease than the BoNT-A side, without complete recovery at 1 month [100]. For EDC, jitter increased at 14 days after a first injection of BoNT-A, peaking between 14 and 30 days after a second double injected dose [98]. At distance, jitter decreased first in the farthest muscles from the injection site [94] (Table 1 and Table S2 in the Supplementary Materials).

When adverse effects were observed after BoNT-A treatment for spasticity (e.g., transient weakness, dysphagia, dysarthria), SFEMG showed an increased jitter in distant non-injected muscles (EDC and/or OO) [90,106] at around 1 month after injection [106] (Table 1 and Table S2 in Supplementary Materials). In the field of esthetics [109,110], the spread of a first injection of BoNT-A to the neighboring muscles was confirmed by an increased jitter within the OO muscle at 2 weeks of treatment [109] and in the contralateral frontalis muscle compared to baseline (*p* = 0.05) [110].

#### 2.2.3. Clinical Correlation and Jitter

The clinical outcome equally comprises evaluation of treatment efficacy and any adverse effects. A significant (*p* < 0.02) improvement of BSP symptoms was seen between the first and the second weeks after BoNT-A injection, with jitter significantly higher than the normal pre-treatment value (*p* < 0.0005), estimated at three times higher than the upper limit of normal value (Table S1 in Supplementary Materials) [101]. Indeed, there was an apparent correspondence between the maximum jitter value and the best clinical outcome [100]. Jitter remained at twice pre-injection value before the recurrence of symptoms [101] (Table 1 and Table S2 in Supplementary Materials). At 1 month, clinical effects reduced while jitter decreased, without a complete recovery [100] (Table 1 and Table S2 in the Supplementary Materials). For those whose BSP symptoms reappeared at Day 100, a significant decrease in jitter was noted compared to baseline [101]. The reappearance of symptoms is accompanied by a decrease in jitter but precedes its normalization [101], sometimes with jitter values three times pre-injection levels at 4 months after the second injection [94].

Clinical effects of BoNT-A transfusion varied widely, with the mechanism remaining largely unknown, and could lead to adverse effects. Transient generalized muscle weakness [102,103], iatrogenic botulism [90] or myasthenia-like clinical presentation (dysphagia, dysarthria, right-sided ptosis, neck extensor muscle weakness) was seen in patients who received treatment either for cosmetic purposes [115] or therapeutic doses of BoNT-A [102,103] and SFEMG showed widespread electrophysiological abnormalities e.g., an abnormal lengthened jitter in the neighboring [115] or distant muscles [102,103,112] (Table 1). Patients who developed a clinical muscle weakness showed increased jitter values versus asymptomatic patients [109]. The disappearance of adverse effects precedes potential recovery of jitter [106], and persistent adverse effects were correlated to an early high value of jitter [94].

#### 2.2.4. Dose-Effect Relationship

Some authors describe a correlation between the dose of BoNT-A injected and the modifications in jitter [95,105,109,110], while others do not [94,96,97]. Several articles have linked the side-effects post-BoNT-A in BSP [117] and in cervical dystonia [105,118] to the doses injected. Unfortunately, the SFEMG analyses were too rare to establish any correlation with jitter [94]. In the field of esthetics, the authors described a correlation between doses of BoNT-A injected into the frown lines and the lengthening of OO jitter between week 0 and 2. There was a trend of increasing jitter values at 2 weeks with higher doses of BoNT-A (10 and 20 units) compared to low doses (5 units) [109]. Conversely, no relationship was found between variations of dose and jitter values [97,99,110], especially if a single dose of BoNT-A was used [96,108].

#### 2.2.5. Outliers

This narrative review has raised questions about some atypical application of SFEMG. In the absence of concrete assays to interpret the duration of effect, occurrence of abnormal blockage and sprouting of NMJ, SFEMG was used to compare treatments, sometimes between different types of BoNT-A [105] or between different BoNT serotypes [116]. Likewise, SFEMG was used to detect early signs of BoNT-A spread assessing the NMJ transmission at distance. Thus, some authors claimed an absence of diffusion if they did not detect a modification of jitter in the muscles distant from the point of injection (EDC, OO) and also confirmed its safety for use in certain fields of application, like the management of morbid obesity [107] or in bladder-sphincter disorders [108] (Table 1).

## 3. Discussion

SFEMG appears to be sensitive to early modifications of the NMJ following BoNT-A injection. Most publications noted very early jitter increase (Day 7 post-injection or even earlier [94]), with a peak between Day 15 and 30 on average [94,96,97,99,109,110]. Yet it is premature to consider jitter as an accurate, reliable [60] and reproducible [119,120] tool considering the large variety of protocols, the arbitrary determination of threshold values (Table S1 in the Supplementary Materials) and the unclear correlation with the clinical evolution. Jitter initially lengthened when symptoms diminished or even disappeared. The reappearance of symptoms accompanies the tendency for jitter to decrease but always precedes its normalization, which is either delayed or nonexistent. Most frequently, the reappearance of symptoms justifies the renewal of BoNT-A treatment once the muscles involved have been identified and evaluated. Jitter may therefore be appropriate when clinical exam is insufficient to assess which muscles are involved and when the muscle appears to be resistant to BoNT-A treatment. In such uncertain situations, the persistence of a strong pathological lengthening of the jitter could question the relevance of reinjection and modify the therapeutic management. Anomalies of the NMJ have been noted at distance from the injected site according to jitter results [95,96,97,98,99] and also in placebo side when contralateral BoNT-A injection was made [94,100]. The time course with which the abnormalities developed and cleared, as well as the kinetics of jitter modifications in the abnormal muscle, indicated that BoNT-A caused the abnormalities in distant muscles studied by SFEMG. Although BoNT-A treatment is considered safe, regional diffusion of the toxin may be responsible for both benign and serious adverse events [102,103,115], supporting the hypothesis of BoNT-A central mediation [121]. The mechanisms underlying this phenomenon of spreading are poorly understood and controversial, possibly involving parameters like dose, volume, and concentration of BoNT-A [122]. A direct action of this toxin via transported hematogenous nerves [123], or by an indirect action leading to “reorganization” of the central nervous system [106,124,125] has been hypothesized in humans and animals [126,127,128,129], but the mechanisms remain unclear. Similarly, questions over a potential residual effect on the motor end plate were raised considering that pathological jitter was found before treatment in a population with a history of previous BoNT-A injections [94,106]. Indeed, the persistence of changes of NMJ after, and at distance from, a BoNT-A injection has been demonstrated in numerous studies in animals and humans [130,131,132,133]. Although the contribution of the neural sprouts to functional recovery is still unclear, two functional steps have been proposed: the first step in which early nerve sprouting induces reappearance of vesicles containing ACh in the nervous terminal and initial muscle contraction; and a second stage of functional recovery restoring the original junctional NMJ followed by regression of initial sprouts [133]. This probably explains the initial trend to normalization of jitter seen prior to the normalization itself. These data are in agreement with reports on histopathological studies [130,134,135,136] analyzing neurogenic atrophy after BoNT-A and the reorganization of MUs developing thereafter [133,137,138,139], with possible modification and atrophy of fibers I and II [106]. We highlighted arguments suggesting a possible dose-effect between increased jitter and BoNT-A dose, especially when the jitter measured was 10 times greater than the initial values [94]. Nevertheless, more powerful studies are required to confirm these findings.

So far, no other literature review has included SFEMG as a tool for evaluating outcomes following injection of BoNT-A. However, it is essential to characterize the effects of this treatment as it provokes a transitory interruption in neuromuscular transmission and a precise evaluation of its therapeutic efficacy is necessary to guarantee optimal management. Although SFEMG seems to be a pertinent tool for spasticity evaluation, its application for involuntary spasms follow-up is questionable, as clinical evaluation seems to be usual [1,3]. Development of more complex clinical scales are proposed [140] with a combination of objective and subjective rating scales and taking into account the impact on activities of daily living [141]. Any biomechanical or electronic measurement device should allow evaluation of spasms based on video analysis. Software has already been described such as machine learning techniques that enable automatic localization of facial landmarks using large datasets of facial photographs [10]. Currently, in the field of spasticity [15,23,24], validity and objectivity [15,20,25,26,27] of current ordinal spasticity scales are controversial, explaining the need for other tools. Other neurophysiological parameters were used to characterize spasticity, but only either occasionally [43,44,142] or with later modifications [48,49] but never BoNT-A treatment throughout. M and H waves have been presented as being pertinent and reliable over the course of treatment with BoNT-A [45], but these measures require standardized methods [143,144]. According to a systematic review, the combined use of instrumental (electrophysiological with the H wave and tonic stretch reflex threshold, pendulum test, strength measurement) and clinical tools (MAS, Tardieu Scale, multiple-item scales) for evaluating spasticity following a stroke is not reliable [20]. Furthermore, ultrasound scanning [36,145,146,147,148] and elastography [39,41,42,149,150,151] allow evaluation of the biomechanical and ultra-structural characteristics of muscle: length, fiber thickness, pinnation angle; with all parameters inversely correlated to the echogenicity. However, these parameters are difficult to assess over the time with a quantitative value. Further studies will be necessary to determine their role in therapeutic follow-up. SFEMG modifications caused by BoNT-A seem to be detectable earlier than ultrasound and elastography but lack a precise threshold. However, the interpretation of the results should consider that jitter may lengthen in the older population as the risk of muscle loss or physiological neuropathy increases with aging. 

A narrative review was performed since investigators had no clear question for a systematic review and considering that the included studies had very moderate statistical power due to the low number of subjects and the limited application of statistical methods. The narrow field under study forced us to include a fairly small number of studies, and most often with observational descriptive design and rarely interventional, randomized or controlled, with a high diversity of protocols, neurological disorders [106,111,115], neuro-muscular districts [97,105,106,108,111], doses and type of BoNT-A [94,95,97,99,102,103], recording times after exposure and follow-up [94,95,96,97,103,108,115]. Sometimes, studies were only partially performed or described [90,94,102,103,104,105,108], and the data obtained were heterogeneous and occasionally contradictory. The recording conditions, such as the technique used, also differed between studies (spontaneous versus stimulated), with a frequent lack of precision of the upper limit of jitter normal value accepted (see US in Table S1 in the Supplementary Materials), preventing comparison. Certain authors preferred the stimulated technique, considered to be more sensitive and simpler, not requiring any patient participation but leading to more artefacts. Again, repeated measurements could cause micro-traumatisms and falsely lengthen jitter or increase the number of artefacts, a weakness that was rarely emphasized in the studies. Follow-up was sometimes insufficient to determine the effect of BoNT-A on the NMJ at a distance. Additionally, certain studies gave no information about the electrophysiological parameters prior to treatment with BoNT-A, notably the existence or not of previous anomalies of the NMJ. The natural variability factors of jitter (the muscle studied, patient age, skin temperature or electrode placement) were not always specified. Very few studies described the means used to control these factors, such as changing sides [99]. A similar standardized procedure for each study would have allowed us to make a fairer comparison. 

Considering the repetitive nature of BoNT-A treatment in the fields of neuromuscular disorders (spasticity and dystonic disorders) and its residual effect with partly reversible modifications in neuromuscular transmission, it is imperative to develop reliable tools to provide objective follow-up. SFEMG appears to fulfil this demand at least partially. Focusing on spasticity treatment, in the majority of cases BoNT-A is renewed every three months, although complete restauration of the NMJ is uncertain [130,152] as recovery of jitter was not expected before treatment was repeated. Before deciding when or if to repeat injections, particularly for cases with unclear clinical treatment efficacy or when treatment becomes inefficient, treatment could be guided by jitter values. In the long-term, this might help to avoid increasing doses that have become inefficient on denervated muscle and to better distribute this maximum dose allowed within the target muscles, thus limiting side-effects and cost. To confirm the hypotheses raised here, the first step would be to design a study on the kinetics of jitter in the injected muscle. It would be pertinent to study patients by groups in comparison to healthy subjects: first with patients never having been injected to provide additional data about BoNT-A naïve hemiplegic muscle [153,154] and then with a multi-injected patient group, providing more information on the residual effect. Secondly, SFEMG will allow physicians to make data-based decisions regarding the best BoNT-A treatment protocol (injected muscle, dose, timing of injection) according to the reference given by muscle and age [59]. The objective is to determine jitter thresholds alone in order to predict the clinical efficacy of BoNT-A treatment.

## 4. Conclusions

SFEMG is an electromyographic technique, which has revealed the mechanisms of NMJ damage. It appears to be a sensitive tool for analyzing the action of BoNT-A toxin by early increase of jitter in the muscle itself and/or at a distance. This narrative review highlights the potential of this tool for clinicians in pharmacological management with BoNT-A. Future studies will be required to certify the reliability of this tool, to develop a predictive model and establish recommendations for the treatment of each muscle with BoNT-A.

## 5. Materials and Methods

A narrative review of studies reporting the use of SFEMG during BoNT-A treatment was based on the Preferred Reporting Item for Systematic reviews and Meta-Analysis (PRISMA) statement [155].

### 5.1. Identification of Studies

Articles were searched for in Medline/Pubmed and Cochrane Library from the date of database creation until May 2020. The research criteria for single keys were used as follows: “single-fiber” OR “SFEMG” OR “single fiber needle electrode” OR “JITTER” OR “electrophysiology” AND “Botulinum”.

### 5.2. Study Selection

First, two reviewers (HM, WF), independently screened the titles and abstracts to identify the most relevant studies that satisfied the inclusion and exclusion criteria (see below). When it was not possible to decide based on the title and abstract, the full text was analyzed. Discrepancies between the reviewers were resolved initially via discussion, and persistent disagreement was resolved by a third reviewer (AD) who made the final decision.

### 5.3. Eligibility Criteria

Articles were retained if BoNT-A treatment was investigated by SFEMG examination (with traditional electrode or CNE). In a first step, only striated skeletal muscle of the limbs, paretic spastic or healthy (to differentiate changes related to spastic paresis) in humans were targeted. Table 1 summarizes details if there was a precise description of the SFEMG procedure (e.g., voluntary or stimulated SFEMG, type of electrode (old standard or CNE), number of potential pairs recorded, the method for determining the mean consecutive difference, the definition of abnormal values and upper limits of jitter accepted), and/or homogeneity of parameters chosen (timing of analyses, muscles injected and measured). There were no specific inclusion or exclusion criteria concerning demographic characteristics or limitations for length of follow up. Only articles in English or French were retained. 

In order to interpret the results, all the jitter values found in the various articles were analyzed according to the references calculated by a collaborative committee by the Ad Hoc Committee of the AAEM Singe Fiber Special Interest Group, with a determination of an upper 95% normal limit for jitter measurements (standard electrode) and mean MCD [61]. As CNE jitter value differs from the standard SFEMG electrode, updated reference values were used [64,65,156].

## Figures and Tables

**Table 1:** Summary of study characteristics.

Authors	Population Number, Age (Years), Disease	Injected Muscle(s)	Jitter (MCD in µs) Mean Pre-Injection Value/Maximum Post Injection Value
Sanders et al. 1986 [94]	n = 4, age and sex US, BSP- HSF	OO bilaterally	OO: 30/>300; Frontal: 24/>300; EDC: 30/50 BB: US/66
Lange et al. 1987 [95]	n = 5, 42‒62 Torticollis, Dystonia	SCM, Trapezius Temporalis	EDC: 28/65.2
Olney et al. 1988 [96]	n = 6, 31‒64, Torticollis	Cervical	BB: 26/46
Lange et al. 1991 [97]	n = 42, 25‒75, Torticollis	Cervical	EDC: 21/43.6; BB: 21.7/US; TA: 28.8/US; Pb: 25.7/26.5
Girlanda et al. 1992 [98]	n = 5, 25‒64, BSP, HSF, Torticollis	US	EDC: US/40
Garner et al. 1993 [99]	n = 8, 31‒66, HSF, MGS, Perioral Dystonia	Right upper lip Cervical, Left eye, Periorbital	EDC: US/156 TA: US/129
Girlanda et al. 1996 [100]	n = 6, 56‒70, BSP	Around one eye BoNT-A Around the other eye Pb	BoNT-A side: <30/250 Placebo side: 20/48
Bogucki et al. 1999 [101]	n = 16, 41‒84, BSP, HSF, MGS	OO	OO: 22.7 ± 2.5/150
Bakheit et al. 1997 [102]	n = 2, 34‒67, MS, MSA with torticollis	Hamstring, SCM, Splenius, Trapezius	EDC: US/408
Bhatia et al. 1999 [103]	n = 3, 32‒57, Cervical dystonia, Hemidystonia	SCM, Splenius, BB, Brachioradialis, Flexor carpi ulnaris, Adductor pollicis, Flexor hallucis brevis, Flexor digitorum	EDC: US/93.6 BB: US/US
Schweizer et al. 1999 [104]	n = 1, 73, US	Thyroarytenoid	Thyroarytenoid: US/40.70
Tang et al. 2000 [105]	n = 785, 5‒82, BSP, Cervical dystonia, HSF, MGS	EDC	EDC *Botox^®^*: 29.9/43.8 *CBTX-A^®^*: 28.5/46.2
Roche et al. 2008 [106]	n = 4, 25‒59, Hemiplegic, Paraplegia, MS	FDL, FDP, FDS, FRC, FHL, TS	EDC: US/54.2 OO: US/31.31
Osio et al. 2010 [107]	n = 24, BoNT-A group 40.6 ± 3.5, Pb group 45.2 ± 3.7, Obesity	Intragastric	EDC: BoNT-A group: 29.12 ± 4.38/33.1; Pb group: 29.44 ± 3.64/33.1 EDC: US/24.9
Schnitzler et al. 2011 [108]	n = 21, 22‒65, Medullary lesion with neurogenic overactive bladder	Intradetrusor injection	OO: US/25.9 BoNT-A group: 27.6/38.9 Pb group: 28.7/31.7
Alimohammadi et al. 2014 [109]	n = 16, 31‒64, healthy	Glabellar muscle	Contralateral Frontalis: 28/39
Punga et al. 2015 [110]	n = 5, 33‒52, Glabellar frown lines	Corrugator muscle	EDC: US/110.9
Ruet et al. 2015 [111]	n = 5, 30‒77, Detrusor hyperactivity, Hypertonia	Detrusor muscle, Striated muscle responsible for dystonia	OO: US/31.6
Szuch et al. 2017 [112]	n = 1, 72, Parkinson’s disease	EHL, FDL, Gastrocnemius, Peroneus longus, Soleus	EDC: US/66.2 for voluntary SFEMG; 52.7 for stimulated SFEMG
Lispi et al. 2018 [113]	n = 10, 42‒64, Healthy	EDB	EDB: 28 ± 7.5/148.3
Leonardi et al. 2019 [90]	n = 2, 32‒48, CP, MS	Quadriceps, Adductors	Deltoid: US/111.4 EDC: US/75.6
Timmermans et al. 2019 [114]	n = 1, 43, Healthy	Glabellar, Forehead, Lateral canthal rhytids	OO: US/112
Punga et al. 2020 [115]	n = 2, 46‒55, Wrinkles, Migraine	Around OO, Glabellar, Head, Neck	Deltoid: US/25; Frontalis: US/US; OO: US/125
Eleopra et al. 2020 [116]	n = 12, 34‒51, Healthy	ADM	ADM: 25.3/133

ADM: abductor digiti minini; BB: biceps brachialis; BSP: blepharospasm; CBTX-A^®^: Chinese type A botulinum toxin; CP: cerebral palsy; EDB: extensor digitorum brevis; EDC: Extensor digitorum communis; EHL: extensor hallucis longus; FDL: flexor digitorum longus; FDP: flexor digitorum profondus; FDS: flexor digitorum superficialis; FHL: flexor hallucis longus; FRC: flexor carpi radialis; HSF: hemifacial spasm; HSP: Hereditary spastic paraparesis; MGS: Meige Syndrome; MS: multiple sclerosis; MSA: multiple system atrophy; OO: orbicularis muscle; Pb: Placebo; SCM: sternocleidomastoid; TA: tibialis anterior; TS: triceps solear; US: unspecified.

## Data Availability

Not applicable.

## Supplementary Materials

The following are available online at https://www.mdpi.com/article/10.3390/toxins13050356/s1: Table S1: Jitter normal value reference for each study; Table S2: Study characteristics.
