# Supplementary material for: Contribution of Single-Fiber Evaluation on Monitoring Outcomes Following Injection of Botulinum Toxin-A: A Narrative Review of the Literature
Source: Toxins (Basel). 2021 May 17;13(5):356. doi: 10.3390/toxins13050356 (PMC8156529; doi:10.3390/toxins13050356)
Supplement: Supplementary file 1 [file toxins-13-00356-s001.zip › supp update.pdf]

Supplementary Materials: Contribution of Single-Fiber Evaluation on Monitoring Outcomes Following Injection of Botulinum Toxin-A: a Narrative Review of the Literature

Hélène Moron, Corine Gagnard-Landra, David Guiraud and Arnaud Dupeyron

Table S1. Jitter normal value reference for each study.

Authors	OO	Frontal	EDC	BB	TA	Other muscles
**Sanders et al. 1986** [1]	<30	<30	<34	<30	US	
**Lange et al. 1987** [2]	US	US	<34	US	US	
**Olney et al. 1988** [3]	US	US	US	<28.9 ± 10.9	US	
**Lange et al. 1991** [4]	US	US	Reference elsewhere [122]			
**Girlanda et al. 1992** [5]	US	US	No reference, Comparison to baseline value	US	US	
**Garner et al. 1993** [6]	US	US	Reference elsewhere [122]	US	Reference elsewhere [122]	
**Girlanda et al. 1996** [7]	No reference, Comparison to baseline value	US	US	US	US	
**Bogucki et al. 1999** [8]	<50 years: 27<br>≥50 years: 29	US	US	US	US	
**Bakheit et al. 1997** [9]	US	US	< 57	US	US	
**Bhatia et al. 1999** [10]	US	US	US	US	US	
**Schweizer et al. 1999** [11]	US	US	US	US	US	Thyroarythenoid US
**Tang et al. 2000** [12]	US	US	Not precisely defined: "normal before injection", pre-injection value written: 29.9 and 28.5	US	No precision "the changes were similar"	
**Roche et al. 2008** [13]	<30	US	<40	US	US	
**Osio et al. 2010** [14]	US	US	Reference elsewhere [60]	US	US	
**Schnitzler et al. 2011** [15]	<10% of pairs exceeded 30 µs<br>Or mean jitter <19 µs	US	<10% of pairs exceeded 40 µs<br>Or mean jitter <24 µs	US	US	
**Alimohammadi et al. 2014** [16]	<34	US	US	US	US	
**Punga et al. 2015** [17]	US	Contralateral frontalis: US	US	US	US	
**Ruet et al. 2015** [18]	Mean jitter <20 µs<br>Individual jitter <30 µs	US	Mean jitter <25 µs<br>Individual jitter <40 µs	US	US	
**Szuch et al. 2017** [19]	US	US	Mean jitter:<br>Voluntary SFEMG: <43,8<br>Stimulated SFEMG: <25	US	US	

**Lispi et al. 2018** [20]	US	US	US	US	US	Injected EDB and contralateral: US
**Leonardi et al. 2019** [21]	US	US	US	US	US	Deltoid: US
**Timmermans et al. 2019** [22]	US	US	US	US	US	
**Punga et al. 2020** [23]	US	US	US	US	US	Deltoid: US
**Eleopra et al. 2020** [24]	US	US	US	US	US	ADM: US

US: unspecified.

Table S2. Study characteristics.

**Authors**	**Population Age (years)-Sex by patient Number of patient by pathology (n)**	**BoNT-A Brand Name ® Number of injections Dose Previous injection**	**Muscle(s) studied**	**SFEMG Device used Calculation jitter methodology**	**Interval between BoNT-A and SFEMG Jitter MCD in μs; By patient and muscle**
Sanders et al. 1986 [1]	age and sex US<br>BSP n = 4	Botox®<br>1 injection<br>except #1 with<br>2 injections<br>All patients: 12.5 MLU<br>Previous: US	OO<br>EDC<br>Frontal<br>BB	Device: US ;<br>Standard electrode<br>No description	#1<br>D -5; OO: 30; Frontal: 24; EDC: 30<br>D 1: OO: 90; Frontal: 21; EDC: US<br>D 2: OO:106; Frontal: 56; EDC: US<br>D 51: OO: 305; Frontal: 56; EDC: 27<br>D 147: OO: 114; Frontal: 49; EDC: US<br>D 51 after the second one: OO: 123; Frontal; 90; EDC: 50<br>D 158 after the second one: OO: 93; Frontal: 37; EDC: 32<br>#2<br>D 53: OO: 300; Frontalis: >300; EDC: 44<br>D 188: OO: 83; Frontalis: 97; EDC: 33<br>#3<br>D 0: OO: 43; EDC: 30<br>D 3: OO: >300; EDC: 30<br>D 60: OO: US; EDC: 37<br>#4<br>D 42: EDC: 40; BB: 66<br>D 100: EDC: 34; BB: 26
Lange et al. 1987 [2]	#1/51/F<br>#2/58/F<br>#3/42/F<br>#4/49/F<br>#5/62/M<br>Dystonic disorders n=3<br>torticollis n = 1<br>oromandibular dystonia n = 1	Botox® *(MU)*<br>2 injections for #1 and #2<br>#1: 285; 450<br>#2: 372; 687<br>#3: 100<br>#4: 302.5<br>#5: 245<br>First injection	EDC	Standard electrode<br>Peak-to-peak amplitude >200 μV, rise time <300 μs,<br>20 pairs	#1: Before injection: 28<br>2 W after the 1st one: 49.2<br>4 Mt after 2nd one: 41<br>#2: 6 W after the 1st one: 37<br>3 W after 2nd one: 36<br>#3: 2 W after the 1st injection: 27.1<br>#4: 3 W after the 1st injection: 54.2<br>#5: 6 W after the 1st injection: 65.2

Olney et al. 1988 [3]	#1/48/F<br>#2/53/F<br>#3/64/F<br>#4/46/F<br>#5/62/M<br>#6/31/M<br>Torticollis n=6	One injection<br>280 UI<br>First injection	BB	Dantec 1500; 13K87<br>Standard electrode<br>Peak-to-peak amplitude >200 μV, rise time <300 μs, constant shape over time. 50 to 100 consecutive discharges of each 20 pairs of voluntarily activated single muscle fibers; commercially available jitter meter	#1: W 0: 26; W 2: 25; W 4: 29<br>#2: W 0: 28; W 2: 32; W 4: 39<br>#3: W 0: 30; W 4: 27<br>#4: W 0: 27; W 8: 41<br>#5: W 0: 30; W 10: 46<br>#6: W 0: 33; W 12: 46
Lange et al. 1991 [4]	Torticollis n = 42	Botox® n = 23<br>Pb n = 19<br>One injection<br>Doses BoNT-A (U)<br>140, 150, 165<br>(detailed elsewhere [99]<br>First injection	EDC n = 36<br>BB n = 4<br>TA n = 2	Standard electrode<br>Computer programs TECA Mystro and Nicolet Viking<br>Peak-to-peak amplitude >200 μV, rise time <300 μs, constant shape over time. At least 50 consecutive discharges of each 20 pairs of voluntarily activated	Before injection (n = 42)<br>EDC 21-30 (for both groups)<br>BB 21.7<br>TA 28.8<br>Pb 25.7<br>2 W (n = 42)<br>EDC BoNT-A 43.6<br>Pb 26.5 *unchanged from preinjection $p < 0.05$*<br>BB: US<br>TA: US<br>12 W (n = 27)<br>EDC 35.5<br>Pb 24.5<br>BB: US<br>TA: US
Girlanda et al. 1992 [5]	Sex US<br>#1 #2 #3 BSP<br>#4 HSF<br>#5 spasmodic torticollis	Botox®<br>#1 #2 #3 #4 20 U<br>#5 65 U<br>2 sessions of injection with 2nd dosage doubled<br>First injection	EDC	Standard electrode<br>20 pairs<br>Electromyograph BASIS EPM (OTE BIOMEDICA)	D 45 after the 1st injection: 32.6<br>D 45 after 2nd injection: 40<br>D 1, 4, 7, 14, 30 after 1st injection and D 1, 4, 7, 14, 30, 60 and 90 after 2nd injection: US for each patient, data represented on figure
Garner et al. 1993 [6]	#1/46/F/Perioral dystonia<br>#2/40/F/Torticollis<br>#3/31/M/Torticollis<br>#4/66/M/Torticollis<br>#5/39/M/HSF<br>#6/58/F/MGS<br>#7/45/M/Torticollis<br>#8/57/F/Torticollis	First injection<br>*One injection (ng):*<br>#1: 5<br>#2: 22.5<br>#3: 15<br>#4: 20<br>#7: 15<br>#8: 20<br>#5: 2<br>#6: 6	EDC all patients<br>Alternatively, right and left sided muscles<br>*Except #5 only right*<br>+ TA for #1 #7	Standard electrode<br>MEDELEC MS 20<br>Peak-to-peak amplitude >500 μV, rise time less than 250 μs<br>20 fiber pairs	Details given for *maximum MCD*<br>#1: EDC: D 13: 142; TA: D13: 129<br>#2: D 7: 121<br>#3 D: US: *normal*<br>#4: D 6: 54.5<br>#5: D US: *normal*<br>#6: D 11: 50.2<br>#7: EDC: D 7: 66.9; TA: D5: 48<br>#8: D 3: 156<br>Data detailled in figure
Girlanda et al. 1996 [7]	Age US; 3F/3M<br>BSP n = 6	Botox® 20 units<br>One injection<br>No detail about injection past history	Bilateral OO	*Mystro Medelec MS25*<br>Standard electrode<br>20 pairs<br>Electrophysiological investigator blinded to treatment	Baseline: BoNT-A: normal (<30 μs); Pb: around 25 μs, [20μs-30μs] W 1: BoNT-A: mean 75 μs , [50 μs-100 μs], $p < 0.02$; Pb: mean 35 μs [25μs-42μs]; *non-significantly* W 2: BoNT-A: mean 150 μs, [50μs-

					250µs], p < 0.01; Pb: mean 41 µs [32µs-48µs], p < 0.01 Mt 1: BoNT-A: mean 125 µs, [50 µs-175µs], p < 0.02; Pb: between 36 and 38 µs, p < 0.02<br>Data detailled in figures
Bogucki et al. 1999 [8]	#1/45/F/MGS<br>#2/67/F/BSP<br>#3/64/F/HSF<br>#4/71/M/BSP<br>#5/41/F/BSP<br>#6/84/F/BSP<br>#7/47/F/HSF<br>#8/59/F/HSF<br>#9/65/F/HSF<br>#10/58/F/MGS<br>#11/72/F/BSP<br>#12/51/F/BSP<br>#13/73/F/MGS<br>#14/69/F/BSP<br>#15/42/M/BSP<br>#16/63/M/MGS<br>BSP n = 8; MGS n = 4; HSF n = 4	Dysport®<br>120 UI/ muscle<br>One injection<br>Bilateral for BSP<br>One side for HSF<br>First injection	OO	Mk2 Dantec 13K87, Denmark<br>Standard electrode<br>Slight voluntary contraction of OO<br>20 pairs	Before injection: 22.7 ± 2.5; n = 16<br>Early remission (D 7-14; mean 9.9 ± 2.2): 99.9 ± 50.1; n = 15/16<br>Late remission (D 23-57; mean 35.6 ± 11.6): 96.3 ± 35.1; n = 15/16<br>When dystonic movements recurred (D 57-265; mean 116.7 ± 52.5): 59.8 ± 18.8; n = 13/16<br>decrease in jitter was noted compared to pretreatment (p < 0.0005), 1st (p < 0.015) and 2nd (p < 0.002) posttreatment analysis
Bakheit et al. 1997 [9]	#1/67/F/MS<br>#2/34/F/MSA with torticollis	Dysport®<br>#1 250 U for left leg<br>#2 750 U<br>250 in right SCM and left splenius capitis - 500 in left trapezius	EDC	Standard electrode	Interval US<br>#1 ranged from 92.6 to 408<br>#2 US
Bhatia et al. 1999 [10]	#1/45/F/Cervical dystonia<br>#2/57/F/Symptomatic hemidystonia<br>#3/32/F/Symptomatic hemidystonia	Dysport®<br>One injection<br>#1: 650 UI: SCM 300, Left splenius capitus 300, Right splenius capitus 200<br>History of 650 UI Dysport® at 12-14 intervals weeks between November 1989 and 1995<br>#2: Total dose 900 UI Dysport®<br>Previous: March 1989 – October 1991: 30 months of injections, total dose at each visit ranged from 350 -800 UI at intervals of 10-12W<br>#3: History: Since January 1988, at 8 and 10 W intervals with an average dose of 600 UI at each visit<br>October 1990 in addition to usual injections further dose of 200UI	#1<br>1st SFEMG right EDC<br>2nd SFEMG right EDC<br>Deltoid iliopsoas<br>3rd SFEMG right EDC<br>#2<br>Right EDC and BB<br>#3<br>US	Standard electrode<br>#1<br>10 pairs	#1<br>W 2: 93.6<br>Mt 4: No precise data: *excessive jitter*<br>Mt 9: Normal 20.1<br>#2<br>Mt 2: BB: normal; EDC: no data precision: *3/11 pairs showed abnormal jitter*<br>#3<br>Mt2: No data: normal

		given into left gastrocnemius			
Schweizer et al. 1999 [11]	73/Sex US<br>SFEMG studied n = 10<br>BoNT-A injection for spasmodic dysphonia n = 1	periodically injected since 1990<br>dose US	laryngeal muscles	CNE	7 Mt after the last injection: 40.7
Tang et al. 2000 [12]	Patients injected Botox® n=192;<br>CBTX-A® n=593<br>only 40 SFEMG<br>5-82/28F/12M<br>HSF n=17; BSP n = 8<br>MGS Syndrome n = 2<br>Cervical dystonia n = 13<br>Botox® n = 18<br>CBTX-A® n = 22	Botox® 99.4 UI (30-300)<br>CBTX-A 107.3 UI (30-300 UI)	EDC<br>TA	Counterpoint MKII<br>Dantec<br>Standard electrode	Before: normal for both Botox® and CBTX-A®<br>W 2-3: Botox®:<br>EDC 29.9 to 36.2 (+21%, p<0.01); CBTX-A®: EDC 28.5 to 36.4 (+27%, p<0.01<br>W 5-8 and Mt 4-5: Data shown in figure only
Roche et al. 2008 [13]	Patients from a cohort of 187 patients<br>Dysport®® n = 33<br>Botox® n = 233)<br>25-59<br>#1/25/M/MS<br>#2/32/F/Paraplegia<br>#3/42/F/Hemiplegic<br>#4/59/F/Hemiplegic	Dysport®<br>#1 1000 UI<br>#21500 UI<br>#3 1000 UI<br>#4 1000 UI<br>One injection for all<br>Previous:<br>#1 Botox®400 UI/ Dysport®1000 UI<br>#2 Botox®500 UI<br>#3 Botox®300 UI<br>#4 Botox®400 UI / Dysport® 1000 UI	#1 EDC<br>#2 EDC<br>#3 OO<br>#4EDC	Nicolet Viking II electromyography<br>Standard electrode<br>Stimulated technique frequency of 10 Hz<br>Peak-to-peak amplitude > 200 µV, rise time < 300 µs, constant shape over time. 100 consecutive discharges of each 20 pairs of stimulated single muscle fibers	#1: D34: 31.1; Mt 3: 25.2<br>#2: D27: 54.22; Mt 3: 23.8<br>#3: D30: 31.31<br>#4: D31: 33.09
Osio et al. 2010 [14]	BoNT-1 group n = 14<br>Pb group n = 10<br>BoNT-A group M/F = 0.2<br>Pb group M/F = 0.3<br>Obesity n = 24	Botox® 200 UI<br>One injection<br>First injection	Right EDC	Voluntary contraction<br>No data for all devices except the needle: *single fiber needle Oxford Instruments*<br>20 pairs registered<br>Calculation methodology not detailed<br>refer to a bibliographic reference [25]	BoNT-A n = 14/14<br>Pb n = 9/10 (1 refused SF)<br>Pretreatment: BoNT-A 29.12; SD 4.38;<br>Pb 29.44; SD 3.64<br>D 8: BoNT-A 29.59; SD 3.55<br>Pb 27.67; SD 3.84<br>Jitter change (post-pre) 95% confidence interval<br>BoNT-A -1.85; 2.79; *p = 0.674*<br>Pb -5.73; 2.17; *p = 0.330*
Schnitzler et al. 2011 [15]	42.1 ± 14.4; sex US<br>#1/60; #2/29;#3/29; #4/50; #5/63;#6/28 #7/39; #8/56;#9/38; #10/50; #11/22;#12/43; #13/34; #14/64;#15/47;#16/26; #17/23;#18/65; #19/38; #20/57;#21/24<br>8F/13M<br>Medullary lesion with neurogenic overactive bladder n = 21	Botox®<br>300UI<br>One injection<br>First injection n = 2/21<br>N = 19/21<br>Mean number of injections prior to study inclusion: 2.6 ± 1.7 (0-6 injections)<br>Average interval between BoNT-A injections Mt 6 ± 1 (Mt 4-12)	EDC n = 18/21<br>OO n = 3/21: #5; #13; #17	Viking EMG II<br>Stimulated SFEMG<br>Peak-to-peak amplitude >200 µV, rise time <300 µs, constant shape over time,<br>100 consecutive discharges of 20 pairs	EDC 17/18: mean jitter 18.9 µs<br>OO: mean 22.2 µs<br>D 26 ± 8 after injection<br>Details of each patient (D)<br>Details of each patient<br>#1: D 29: 24.90; #2: D30: 15.90; #3: D14: 21.05; #4: D24: 24.17; #5: D50: 25.90; #6: D 25: 19.50; #7: D 15: 21.20; #8: D 21: 23.40; #9: D 24: 15.50; #10: D 30: 19.09; #11: D 17: 22.15; #12: D 34: 37.50; #13: D 34: 20; #14: D 24: 17.17; #15: D 22:

					23.47; #16: D 36: 20.90; #17: D24: 20.8; #18: D 28: 18.45; #19: D 24: 15.60; #20: D 25: 15.20; #21: D 22: 15.30
Alimohammadi et al. 2014 [16]	Age US; 16F/0M<br>Healthy n = 16	*Vistabel*<br>Each group n = 4<br>5 UI<br>10 UI<br>20 UI<br>Pb group<br>One injection<br>First injection	OO	Keypoint Medtronic<br>Slight voluntary contraction,<br>20 pairs	Baseline: mean 28 (23-32) whole group<br>W 2: for 12 intervention subjects, increased to 35 (30-39), p < 0.001<br>Pb: 29<br>-Individual (only detailed for W 0-2)<br>Baseline<br>Pb: Median 28.7; Mean 28.8 ± 1.7<br>5 UI: Median 25.8; Mean 25.8 ± 3.1<br>10 UI: Median 28.1<br>Mean 28.1 ± 0.8<br>20 UI: Median 29.1; Mean 29.1 ± 2.7<br>W 2<br>Pb: Median 29.3; Mean 29.3 ± 2.4<br>5 UI: Median 33.9; Mean 33.9 ± 4.2<br>10 UI: Median 34.4<br>Mean 35.1 ± 2.7<br>20 UI: Median 37.1; Mean 36.4 ± 2.5<br>-Mean change between W 0-2<br>Pb group 1.9 ± 11.6 % *p = 0.76*<br>5 UI group 32.8 ± 23.8 % *p = 0.070*<br>10 UI group 24.9 ± 10.6 *p = 0.018*<br>20 UI group 26.1 ± 16.8 % *p = 0.053*
Punga et al. 2015 [17]	33-52/5F; Sex US<br>Visible glabellar frown lines at rest n = 5	*Vistabel*<br>Onabotulinumtoxin A<br>Total dose : 2.5 UI n= 1;<br>5 UI n = 3;<br>10 UI n = 1;<br>One injection<br>First injection	contralateral frontalis muscle	Keypoint Medtronic<br>20 pairs	28 range 25-32<br>34 range 27-39 *p = 0.05*
Ruet et al. 2015 [18]	Age & Sex US<br>SFEMG n = 35 patients presented side effects after BoNT-A injection, categorized into:<br>Fatigue group n = 15; age 48 ± 14<br>Pseudo-botulism group n = 20; age 41 ± 11<br>Control group n = 17; age 43 ± 13<br>Botulism group n = 3; age 53 ± 24	Botox® / Dysport®<br>Fatigue group 10/4 *<br>Pseudo-botulism group 15/4 *<br>Control group 17/0	EDC/OO<br>Fatigue group 8/7<br>Pseudo-botulism group 11/9<br>Control group 15/2	Neuropack M1<br>20 pairs	Fatigue group: D 36 ± 21<br>EDC 20.5 ± 2.8 (15.0 – 33.1)<br>OO 20.8 ± 3.17 (16.6 – 31.6)<br>Pseudo-botulism group: D 42 ± 24<br>EDC 42.2 ± 24.4 (21.5 - 110.9)<br>OO 21.7 ± 8.7 (13.8 - 31.3)<br>Control group: D 24 ± 8

					EDC 20.9 ± 7.2 (15.2 – 37.5)<br>OO 22.9 ± 2.9 (20 – 25.9)<br>EDC: Jitter significantly higher in the pseudo-botulism group than in the control ($p < 0.001$) and fatigue group ($p = 0.0005$)<br>No difference between fatigue and control group<br>OO: no significant difference between groups
Szuch et al. 2017 [19]	Age & Sex US<br>N = 1<br>right foot dystonia with iatrogenic botulism (head drop, bulbar and systemic weakness in Parkinson's disease	Botox®<br>4 M from previous one<br>Total 600 UI 5th injection:<br>EHL 50; FDL 150<br>Gastrocnemius 150<br>Peroneus longus 150<br>Soleus 100<br>Previous injections:<br>1st and 2nd "preparation and dose unknown"<br>3rd 400 UI time US<br>4th 600 UI 5M later	Left EDC	Nicolet Viking IV<br>Voluntary SFEMG<br>Upper limit 43.8<br>+ Stimulated SFEMG<br>Upper limit 25<br>Methodology not detailed	Voluntary SFEMG: D 21<br>8 pairs with mean jitter 66.24<br>(norm set at 43.8 µs)<br>Stimulated SFEMG: D 21<br>16 pairs with mean 52.74<br>(norm set below 25 µs)
Lispi et al. 2018 [20]	Age US; 2F/8M<br>mean 53<br>Healthy n = 10	Incobotulinuntoxin A Merz®<br>15 UI each patient<br>One injection<br>First injection	EDB bilaterally	Medelec Synergy using a concentric needle *described elsewhere* [26]	Baseline: 28 ± 7.5<br>W1: 33 ± 6.7<br>W2: 45 ± 9.2<br>W3: 55 ± 8.1<br>W4: 111 ± 10.1<br>W 6: 120 ± 9.5<br>W 8: 142 ± 6.3<br>W 10: 110 ±11.3<br>W 12: 66 ± 8.5
Leonardi et al. 2019 [21]	#1/48/F/MS<br>#2/32/M/CP	#1 Botox®: 400 UI<br>2 Mt later: 600 UI<br>5 Mt later: 400 UI<br>#2 : Dysport®: 1500 UI	#1 left deltoid<br>#2 EDC	US	#1: Mt 5: 111.4<br>#2: D 10: 75.6
Timmermans et al. 2019 [22]	43/M	Azzalure®<br>84 UI<br>Previous<br>*Similar injection* 2 years earlier	Left OO	US	W 6: 112
Punga et al. 2020 [23]	#1/55/F/wrinkles<br>#2/46/M/migraine	#1<br>Dose: no data<br>Previous injections<br>*Also received regular BONT-A injections at a beauty clinic*<br>#2<br>14 treatments of 155 UI of Dysport® *divided between a total of 31 points (≈3UI/injection point)*	#1<br>Left OO<br>Left frontalis<br>Left Deltoid<br>#2<br>Left OO	CNE<br>Voluntary technique<br>No device cited	#1: 1 Mt and *a few days*<br>OO 125 µs, *60% increased jitter*,<br>Frontalis: No precise data: *increased jitter 70%*<br>Mt 2: OO: 62 µs;<br>Frontalis: *jitter was increased*<br>Deltoid 25 µs, normal<br>#2: W 2: OO: 37 µs; *increased jitter 38%*<br>Mt 4: *Jitter analysis in the left OO […] also normal*

Eleopra et al. 2020 [24]	N = 12<br>(24 ADM)<br>7M / 5F<br>randomly assigned according to a randomized plan<br>single blind (patient)	First injection for all BoNT-A, B, C, F and placebo<br>5 ADMs 15 U of BoNT-A (Botox®)<br>5 ADMs 1500 U of BoNT-B<br>ADMs 15 U of BoNT-C<br>5 ADMs 15 U of BoNT-F<br>5 ADM 0.3 mL of saline solution (placebo)<br>BoNT/A vs BoNT/B n = 2<br>BoNT/F vs BoNT/C n = 2<br>BoNT/B vs BoNT/F n = 1<br>BoNT/ B vs BoNT/C n = 1<br>BoNT/A vs BoNT/F n = 1<br>BoNT/A vs BoNT/C n = 1<br>each BoNT serotype (A, B, C or F) vs placebo n = 4	ADM	Expressed in MSD *mean standard deviation* , µs<br>SFEMG MSD (mean ± 2.5 SD)<br>Keypoint device (Medtronic®)	Before<br>BoNT-A 25.5 (13)<br>BoNT-B 25.5 (11)<br>BoNT- C 24.5 (12)<br>BoNT-F 25 (12)<br>Placebo 26 (13)<br>W 2<br>BoNT-A 89 (35)<br>BoNT-B 78 (38)<br>BoNT- C 80 (30)<br>BoNT-F 108 (25)<br>Placebo 28 (14)<br>W 4<br>BoNT-A 72.5 (25)<br>BoNT-B 69 (18)<br>BoNT- C 69 (26)<br>BoNT-F 90 (30)<br>Placebo 30 (11)<br>W 6<br>BoNT-A 67 (19)<br>BoNT-B 71 (22)<br>BoNT- C 65 (27)<br>BoNT-F 84 (29)<br>Placebo 24 (15)<br>W 8<br>BoNT-A 52 (22)<br>BoNT-B 63 (28)<br>BoNT- C 55 (18)<br>BoNT-F 51 (20)<br>Placebo 27 (13)

In italics: cited from original text#: Patient; *: missing data; ADM: abductor digiti minini; BB: biceps brachialis; BSP: blepharospasm; CBTX-A®: Chinese type A botulinum toxin; CP: cerebral palsy; D: day; EDB: extensor digitorum brevis; EDC: Extensor digitorum communis; EHL: extensor hallucis longus; F: female; FDL: flexor digitorum longus; FDP: flexor digitorum profondus; FDS: flexor digitorum superficialis; FHL: flexor hallucis longus; FRC: flexor carpi radialis; HSF: hemifacial spasm; HSP: Hereditary spastic paraparesis; M: male; MCD: Mean consecutive difference; MGS: Meige Syndrome; MLU: Mouse lethal unit; MS: multiple sclerosis; MSA: multiple system atrophy; Mt: Month; MU: Mouse unit: 2.5 ng of Botox® = 1 U = mouse LD50 (99); OO: orbicularis muscle; Pb: placebo; SCM: sternocleidomastoid; SD: standard deviation; TA: tibialis anterior; TS: triceps solear; UI: international unit; US: unspecified; W: week.

References

1. Sanders, D.B.; Massey EW; Buckley EG. Botulinum toxin for blepharospasm: Single-fiber EMG studies. *Neurol* **1986**, *36*, 545–547.
2. Lange, D. J., Brin, M. F., Warner, C. L., Fahn, S., Lovelace, R. E. Distant effects of local injection of botulinum toxin. *Muscle Nerve* **1987**, *10*, 552–555.
3. OIney, R. K., Aminoff, M. J., Gelb, D. J., Lowenstein, D. H. Neuromuscular effects distant from the site of botulinum neurotoxin injection. *Neurol* **1988**, *38*, 1780–1783.
4. Lange, D.J.; Rubin, M.; Greene, P.E.; Kang, U.J.; Moskowitz, C.B.; Brin, M.F.; Lovelace, R.E. Fahn,S., Distant effects of locally injected botulinum toxin: A double-blind study of single fiber EMG changes. *Muscle Nerve* **1991**, *14*, 672–675.
5. Girlanda, P.; Vita, G.; Nicolosi, C.; Milone, S.; Messina, C. Botulinum toxin therapy: Distant effects on neuromuscular transmission and autonomic nervous system. *J Neurol Neurosurg Psychiatry* **1992**, *55*, 844–845.
6. Garner, C.G.; Straube, A.; Witt, T.N.; Gasser, T.; Oertel, W. H. Time course of distant effects of local injections of botulinum toxin. *Mov Disord Off J Mov Disord Soc* **1993**, *8*, 33–37.

7. Girlanda, P.; Quartarone, A.; Sinicropi, S.; Nicolosi, C.; Messina, C. Unilateral injection of botulinum toxin in blepharospasm: Single fiber electromyography and blink reflex study. *Mov Disord Off J Mov Disord Soc* **1996**, *11*, 27–31.
8. Bogucki, A. Serial SFEMG studies of orbicularis oculi muscle after the first administration of botulinum toxin. *Eur J Neurol* **1999**, *6*, 461–467.
9. Bakheit, A.M.; Ward, C.D.; McLellan, D.L. Generalised botulism-like syndrome after intramuscular injections of botulinum toxin type A: A report of two cases. *J Neurol Neurosurg Psychiatry* **1997**, *62*, 198.
10. Bhatia, K.P.; Münchau, A.; Thompson, P.D.; Houser, F.; Chauhan, V.S.; Hutchinsin, M.; Shapira, A.H.V.; Marsden, C.D. Generalised muscular weakness after botulinum toxin injections for dystonia: A report of three cases. *J Neurol Neurosurg Psychiatry* **1999**, *67*, 90–93.
11. Schweizer, V.; Woodson, G.E.; Bertorini, T.E. Single-fiber electromyography of the laryngeal muscles. *Muscle Nerve* **1999**, *22*, 111–114.
12. Tang, X.; Wan, X. Comparison of Botox with a Chinese type A botulinum toxin. *Chin Med J (Engl)* **2000**, *113*, 794–798.
13. Roche, N.; Schnitzler, A.; Genêt, F.F.; Durand, M. C.; Bensmail, D. Undesirable distant effects following botulinum toxin type a injection. *Clin Neuropharmacol* **2008**, *31*, 272–280.
14. Osio, M.; Mailland, E.; Muscia, F.; Nascimbene, C., Vanotti, A., Bana, C.; Corsi; F.; Foschi, D.; Mariani; C. Botulinum neurotoxin-A does not spread to distant muscles after intragastric injection: A double-blind single-fiber electromyography study. *Muscle Nerve* **2010**, *42*, 165–169.
15. Schnitzler, A.; Genet, F.; Durand M-C.; Roche, N.; Bensmail, D.; Chartier-Kastler, E.; Denys, P. Pilot study evaluating the safety of intradetrusor injections of botulinum toxin type A: Investigation of generalized spread using single-fiber EMG. *Neurourol Urodyn* **2011**, *30*, 1533–1537.
16. Alimohammadi, M.; Andersson, M.; Punga, A.R. Correlation of botulinum toxin dose with neurophysiological parameters of efficacy and safety in the glabellar muscles: A double-blind, placebo-controlled, randomized study. *Acta Derm Venereol* **2014**, *94*, 32–37.
17. Punga, A.R.; Eriksson, A.; Alimohammadi, M. Regional diffusion of botulinum toxin in facial muscles: A randomised double-blind study and a consideration for clinical studies with split-face design. *Acta Derm Venereol* **2015**, *95*, 948–951.
18. Ruet, A.; Durand, M.C.; Denys, P.; Lofaso, F.; Genet, F.; Schnitzler, A. Single-fiber electromyography analysis of botulinum toxin diffusion in patients with fatigue and pseudobotulism. *Arch Phys Med Rehabil* 2015; 96, 1103–1109.
19. Szuch, E.; Caress, J.B.; Paudyal, B.; Brashear, A.; Cartwright, M. S.; Strowd III, R. E. Head drop after botox: Electrodiagnostic evaluation of iatrogenic botulinum toxicity. *Clin Neurol Neurosurg* **2017**, *156*, 1–3.
20. Lispi, L.; Leonardi, L.; Petrucci, A. Longitudinal neurophysiological assessment of intramuscular type-A botulin toxin in healthy humans. *Neurol Sci Off J Ital Neurol Soc Ital Soc Clin Neurophysiol* **2018**, *39*, 329–332.
21. Leonardi, L.; Haggiag, S.; Petrucci, A.; Lispi, L. Electrophysiological abnormalities in iatrogenic botulism: Two case reports and review of the literature. *J Clin Neurosci Off J Neurosurg Soc Australas* **2019**, *60*, 138–141.
22. Timmermans, G.; Depierreux, F.; Wang, F.; Hansen, I.; Maquet, P. Cosmetic Injection of Botulinum Toxin Unmasking Subclinical Myasthenia Gravis: A Case Report and Literature Review. *Case Rep Neurol* **2019**, *11*, 244–251.
23. Punga, A.R.; Liik, M. Botulinum toxin injections associated with suspected myasthenia gravis: An underappreciated cause of MG-like clinical presentation. *Clin Neurophysiol Pract* **2020**, *5*, 46–49.
24. Eleopra, R.; Rinaldo, S.; Montecucco, C.; Rossetto, O.; Devigili, G. Clinical duration of action of different botulinum toxin types in humans. Toxicon Off *J Int Soc Toxinology*. Epub ahead of print 14 March 2020, doi:10.1016/j.toxicon.2020.02.020.
25. Ekstedt, J.; Nilsson, G.; Stalberg, E. Calculation of the electromyographic jitter. *J Neurol Neurosurg Psychiatry* **1974**, *37*, 526–539.

26. Benatar, M.; Hammad, M.; Doss-Riney, H. Concentric-needle single-fiber electromyography for the diagnosis of myasthenia gravis. *Muscle Nerve* **2006**, *34*, 163–168.
